# Exploring patterns of beta‐diversity to test the consistency of biogeographical boundaries: A case study across forest plant communities of Italy

**DOI:** 10.1002/ece3.5669

**Published:** 2019-10-02

**Authors:** Alessandro Chiarucci, Juri Nascimbene, Giandiego Campetella, Stefano Chelli, Matteo Dainese, Daniele Giorgini, Sara Landi, Chiara Lelli, Roberto Canullo

**Affiliations:** ^1^ Department of Biological, Geological and Environmental Sciences University of Bologna Bologna Italy; ^2^ Plant Diversity and Ecosystems Management Unit School of Biosciences and Veterinary Medicine University of Camerino Camerino Italy; ^3^ Institute for Alpine Environment Eurac Research Bolzano Italy; ^4^ Department of Natural and Land Sciences University of Sassari Sassari Italy

**Keywords:** beta‐diversity, biogeographical regionalization, forest plant communities, ICP Forests, Italy, nestedness, turnover

## Abstract

**Aim:**

To date, despite their great potential biogeographical regionalization models have been mostly developed on descriptive and empirical bases. This paper aims at applying the beta‐diversity framework on a statistically representative data set to analytically test the consistency of the biogeographical regionalization of Italian forests.

**Location:**

Italy.

**Taxon:**

Vascular plants.

**Methods:**

Forest plant communities were surveyed in 804 plots made in a statistically representative sample of forest communities made by 201 sites of Italian forests across the three biogeographical regions of the country: Alpine, Continental, and Mediterranean. We conducted an ordination analysis and an analysis of beta‐diversity, decomposing it into its turnover and nestedness components.

**Results:**

Our results provide only partial support to the consistency of the biogeographical regionalization of Italy. While the differences in forest plant communities support the distinction between the Alpine and the other two regions, differences between Continental and Mediterranean regions had lower statistical support. Pairwise beta‐diversity and its turnover component are higher between‐ than within‐biogeographical regions. This suggests that different regional species pools contribute to assembly of local communities and that spatial distance between‐regions has a stronger effect than that within‐regions.

**Main conclusions:**

Our findings confirm a biogeographical structure of the species pools that is captured by the biogeographical regionalization. However, nonsignificant differences between the Mediterranean and Continental biogeographical regions suggest that this biogeographical regionalization is not consistent for forest plant communities. Our results demonstrate that an analytical evaluation of species composition differences among regions using beta‐diversity analysis is a promising approach for testing the consistency of biogeographical regionalization models. This approach is recommended to provide support to the biogeographical regionalization used in some environmental conservation polices adopted by EU.

## Graphical abstract

This paper aims at applying the beta‐diversity framework on a statistically representative data set to test the consistency of the biogeographical regionalization of Italian forests based on the three different regions recognised within the country. Our results provide only partial support to the consistency of the biogeographical regionalization of Italy based on three regions. Our findings support the existence of a biogeographical structure of the species pools that is captured by the biogeographical regionalization. However, nonsignificant differences between the Mediterranean and Continental biogeographical regions suggest a weakness in this biogeographical regionalization.

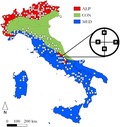

## 1 INTRODUCTION

A biogeographical regionalization is a hierarchical zonation system based on the distribution patterns of the biota, reflecting the pool of species that within each region contribute to the assembly of local communities (Carstensen, Lessard, Holt, Borregaard, & Rahbek, 2013; Cox, Moore, & Ladle, 2016). While the scientific validity of this approach is under debate (Morrone, 2018), the biogeographical regionalization is pragmatically used by biogeographers, ecologists, and conservation biologists for exploring and interpreting biogeographical patterns of species and communities across the world (Ordynets et al., 2018), for providing the basis for conservation priority‐setting and planning (e.g., Fenu et al., 2017; Kreft & Jetz, 2010; Rueda, Rodriguez, & Hawkins, 2010), and for sampling stratification in monitoring plans (Jongman et al., 2006). Moreover, the intuitiveness of this approach facilitates the communication of biogeographical concepts to a nonspecialist such as policy makers (e.g., Campagnaro, Trentanovi, & Sitzia, 2018), providing them a tool for setting up conservation or management policies.

Despite their great potential to advance both theoretical and applicative disciplines, biogeographical regionalization models have been largely developed on descriptive and empirical bases (Olson et al., 2001; Posadas, Grossi, & Ortiz‐Jaureguizar, 2013), but only rarely tested analytically (Kreft & Jetz, 2010; but see Procheş, 2005) to quantify compositional differences among regions. However, in the last decade the increasing availability of species distribution and phylogenetic data coupled with the development of new methodological approaches (e.g., Kreft & Jetz, 2010) have fostered analytical tests at both global and regional scales (Ficetola, Mazel, & Thuiller, 2017; Holt et al., 2013; Rueda et al., 2010; Vilhena & Antonelli, 2015). Thus, it is expected that these new developments can further improve the accuracy of biogeographical regionalization models. In this context, evaluating differences in species composition among regions is one of the most promising tools to test the consistency of biogeographical regionalization models (Morrone, 2018).

Besides traditional multivariate analyses of species composition (e.g., clustering, ordination), comparisons can be made in terms of beta‐diversity, i.e., the variation in species composition among sites in a geographical region (Baselga, Gómez‐Rodríguez, & Lobo, 2012; González‐Orozco et al., 2014). Beta‐diversity can be decomposed into two components—turnover and nestedness of species assemblages—which reflect different processes of community organization (e.g., Baselga, 2010; Carvalho, Cardoso, & Gomes, 2012; Legendre, 2014). The turnover component describes a replacement of species from one site to another, while the nestedness component describes how species‐poor sites are a subset of species‐rich sites (Atmar & Patterson, 1993; Baselga, 2012) Exploring the different components of beta‐diversity across and within biogeographical regions may provide substantial information on the consistency of biogeographical regionalization (Baselga et al., 2012; González‐Orozco et al., 2014). For instance, while both components of beta‐diversity are expected to contribute to the patterns of local community assembly (Baselga, 2012), it is likely that turnover is higher between different biogeographical regions than within the biogeographical regions, reflecting differences in the regional species pool. Conversely, nestedness might be higher within the biogeographical regions, reflecting instead local patterns of species richness within a well delimited species pool.

Here, using a statistically representative sample of forest communities across Italy, we applied the beta‐diversity framework coupled with the analysis of species composition to test the consistency of the biogeographical regionalization of Italian forests, divided by the EU into three different regions: Alpine, Continental, and Mediterranean. Despite its cardinal role in driving the EU policies for nature protection, this biogeographical regionalization is mainly based on an expert interpretation of the digital version of the “Map of Natural Vegetation” (ETC‐BD, 2006). Analytical tests that verify its accuracy are virtually lacking (but see Fekete, Király, & Molnár, 2016 for the Pannonian region). Therefore, testing the consistency of this biogeographical regionalization could provide a dynamic, science‐based framework for better achieving conservation tasks (Morrone, 2018). In this perspective, Italy is a unique model system, spanning a huge latitudinal, climatic, and topographic gradient associated with complex historical and evolutionary scenarios that resulted in a high plant diversity (Svenning, Fløjgaard, & Baselga, 2011), making this country one of the core areas of the Mediterranean biodiversity hotspot. For this purpose, we focused on forest vegetation that in Italy accounts for 35% of the target habitats for biodiversity conservation according to EU policies (Genovesi et al., 2014). In addition, forests provide an interesting study system since they are distributed across the three biogeographical regions, covering 36% of the country (INFC, 2005), and are habitats with a relatively high degree of naturalness.

Based on this framework, we expect a consistent biogeographical regionalization of the Italian forests if the compositional heterogeneity (i.e., beta‐diversity) will be higher between than within the biogeographical regions (e.g., Barton et al., 2013). This would suggest differences in the regional species pool among the biogeographical regions. This result will be further reinforced if the turnover component will also be higher between than within the biogeographical regions, reflecting the fact that species are replaced between the different biogeographical regions. Conversely, nestedness should show an opposite pattern that would suggest that local patterns of species richness are more important in structuring communities within each region. In contrast, we expect no consistent biogeographical regionalization if significant differences or even higher beta‐diversity will be found within‐ than between‐regions. This would indicate that the compositional heterogeneity within each region does not differ, or is higher than between‐regions, and that current biogeographical boundaries do not reflect the separation of different regional species pools determined by biogeographical processes.

## 2 METHODS

### 2.1 Sampling design and data collection

**Figure 1:**
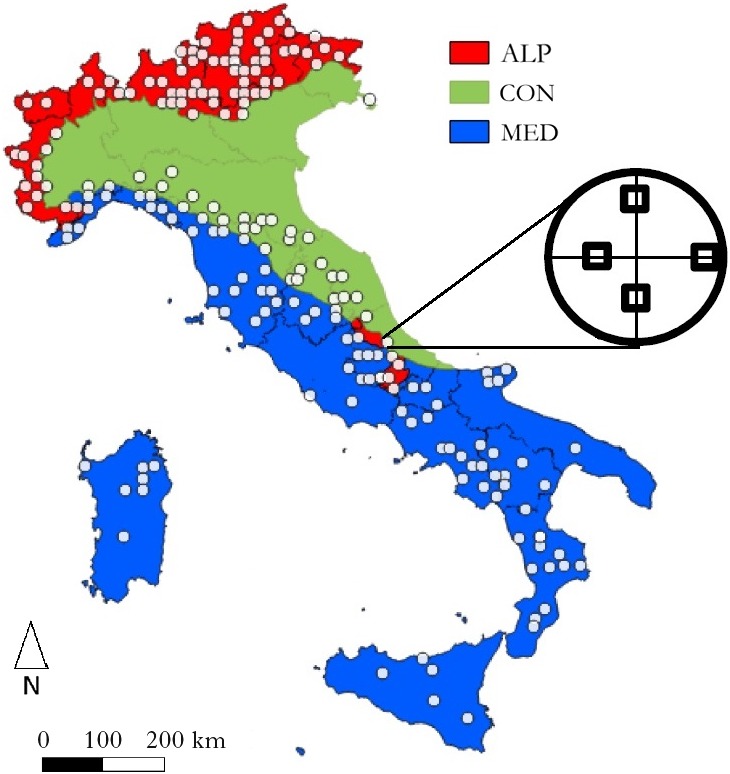
Location of the 201 sampling sites. In each sampling site, 4 10 m × 10 m plots were located at a random distance between 11 and 25 m from the center of the sampling point along the NE, SE, SW, and NW directions. This resulted in 804 plots within 201 sites. The occurrence of all vascular plants was recorded in each plot, and these data were used to produce a species by plot matrix. ALP, Alpine region; CON, Continental region; MED, Mediterranean region

The study area is the whole territory of Italy, which is 301,340 km^2^, with all the forests growing there as a target statistical population. Data were collected in the framework of the BIOSOIL project (Hiederer & Durrant, 2010) using a probabilistic sample of the existing plant communities (Chiarucci, 2007; Lájer, 2007). The BIOSOIL sampling design was based on a 16 km × 16 km grid superimposed on the whole country (Level I network: ICP Forests, 2016; Lorenz et al., 2002); then, each corner of this grid was selected as a sampling site if a forest habitat (larger than 0.01 km^2^) occurred therein. This resulted in a potential sample of 261 sites (Petriccione & Cindolo, 2006), which were used for the field sampling. Being sampled according to a probabilistic approach, these sampling sites are statistically representative of the total forests occupying the entire investigated area (i.e., 87.590 km^2^, 68% of which are deciduous forests, 13% coniferous forests, 10% mixed forest, and 9% not classified forests; INFC, 2005). Sixty sites were not sampled because they were fond not correspond to forests once located on the ground or were inaccessible or extremely disturbed (e.g., cattle rest areas, ski slopes) or were subjected to harvesting operation during the sampling. This resulted into a final number of 201 sampling sites (Figure 1) that was classified into one of three biogeographical regions, as follow: (a) Alpine, ALP; (b) Continental, CON; and (c) Mediterranean, MED.

In each circular sampling site (*r* = 25.24 m, 2,000 m^2^), four 10 m × 10 m plots were located at a random distance, along the NE, SE, SW, and NW directions (Figure 1), resulting in 804 plots. A ground vegetation survey was performed in each plot following a standard protocol (Canullo, Starlinger, & Giordani, 2013; Ferretti et al., 2013). Data on the presence–absence of all vascular plants were used to produce a species by plot matrix. The occurrences of all four plots were combined in a sampling point to describe the species composition of a sampling site. Therefore, we performed all the analyses described below by aggregating the values at site level.

The surveys were carried out in spring‐summer 2007, employing ten teams composed by two surveyors, after a common training and intercalibration exercise following Quality Assurance guidelines (Allegrini, Canullo, & Campetella, 2009; Canullo, Starlinger, Granke, Fischer, & Aamlid, 2016).

### 2.2 Data analysis

First, we conducted a multivariate analysis to assess variation in species composition among the three biogeographical regions. To extract and visualize floristic patterns, we used Non‐metric Multidimensional Scaling (NMDS) using the Jaccard dissimilarity index calculated from the species presence–absence matrix. The 201 sampling units were classified according to biogeographical region of the site location, and the analysis of similarity test (ANOSIM) was applied in order to test differences among regions. ANOSIM is a nonparametric technique that allows statistical comparisons between‐ and within‐groups (Clarke & Green, 1988). ANOSIM tests the significance of the difference between two or more groups of sampling units. It compares the ranks of distances between‐groups with ranks of distances within‐groups. The procedure uses the Jaccard similarity matrix to calculate *R* = (rB−rW)/*N*(*N*−1)/4, where rW is the average of all rank similarity for samples within the same group, rB is the average of all rank similarities for samples between different groups, and *N* is the total number of samples under consideration. *R* varies from +1 to −1. *R* values greater than 0 indicate a higher dissimilarity between‐groups than within‐groups, *R* values equalling zero indicate an equal level of between‐groups and within‐group average dissimilarity. Negative values of *R* indicate that dissimilarities within groups are greater than dissimilarities between groups (Clarke & Green, 1988). Pairwise comparisons of *R* across regions were thus used to test the robustness of the clusters, as follows: sharp separability (*R* > .75); good separability (.5 < *R* ≤ .75); and separated but overlapping (.25 < *R* ≤ .5). These analyses were carried out in the R environment with the vegan package (Oksanen et al., 2015).

Second, the pairwise beta‐diversity was calculated using the Jaccard (1901) dissimilarity index for all the pairs of sites within the same biogeographical region and for all the pairs of sites across different biogeographical regions. To test the significance differences for each comparison, an unpaired *t* test was applied between the pairs calculated within and across biogeographical regions. The different extents of the three biogeographical regions (Dungan et al., 2002) determined differences across the possible pairs of plots. Therefore, to reduce this bias, we limited the analyses to those pairs of plots, both within and between the biogeographical regions, by the maximum extent of 600 km (this represents the extent of the smaller biogeographical region within the study area).

Finally, the turnover and nestedness components of the Jaccard dissimilarity were calculated in order to disentangle the two different processes affecting changes in species composition. Thus, using the equation *ß*_jac_ = *ß*_jtu_ + *ß*_jne_, overall beta‐diversity (*ß*_jac_) can be additively partitioned into two portions representing spatial turnover in species composition (*ß*_jtu_) and variation in species composition due to nestedness (*ß*_jne_). The turnover and nestedness components were calculated using the R function “betapart” (Baselga & Orme, 2012). To test the significance differences (*p* < .05) in each comparison of the total Jaccard dissimilarity (Beta Dissimilarity) and its turnover and nestedness components, an unpaired *t* test was applied between the pairs calculated within and across biogeographical regions. Since we are aware of possible bias due to pseudoreplication due to nonindependence of sites, we tested the robustness of these analyses using independent subsets of sites (i.e., sites that were used for within region calculation were not used for across regions calculation) and repeating the analyses 100 times.

## 3 RESULTS

A total of 1,091 subgeneric *taxa* were recorded in the 201 sites and 804 plots. Most of these *taxa* (hereafter referred to as species) were identified at the species level, while some others were grouped as within subgeneric groups (e.g., in the case of *Rubus* or *Rosa*). The mean number of species per plot was 21.6 (range 2–64), while the mean number of species per site was 38.1 (range 5–111).

**Figure 2:**
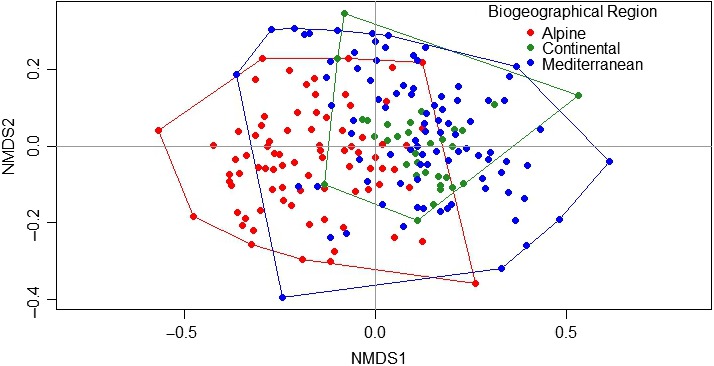
Ordination plot of the 201 sampling sites in the species space based on non‐metric multidimensional scaling (NMDS)

The NMDS ordination had a stress value of 0.225, and the stress versus dimensions‐plot indicated that two dimensions were best suited for representing our results. The visual interpretation of the NMDS plot (Figure 2) indicated a large overlap between plant communities of the Continental and Mediterranean regions, while those of the Alpine region formed more discrete and separated groups in the ordination space. Accordingly, ANOSIM indicated that plant communities of the Alpine region significantly differed from those of the Continental and Mediterranean regions (ALP vs. CON ANOSIM statistic *R* = .2061, *p*‐value = .001; ALP vs. MED ANOSIM statistic *R* = .4461, *p*‐value = .001), while the differences in species composition between the two latter regions were not significant (MED vs. CON ANOSIM statistic *R* = −.02938, *p*‐value = N.S).

**Figure 3:**
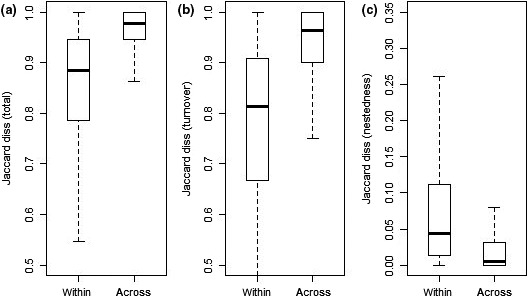
Beta‐diversity, expressed as Jaccard dissimilarity (1‐J) for all pairs of plots within and across biogeographical regions (a). Nestedness and turnover components of the Jaccard dissimilarity (1‐J) for all pairs of plots within and across biogeographical regions (respectively, b and c). To control for differences in extent, only pairs of plots within extent <600 km were included in the analyses. The horizontal bold line represents the median, the boxes lines extremes represent the 25th and 75th percentile while whiskers represent the 1th and 99th percentiles

Beta‐diversity was significantly higher across biogeographical regions than within biogeographical regions (Figure 3a, *t* = 20.8316, *p* < .001). The turnover component of beta‐diversity contributed the majority of the total variation, and it was significantly higher across than within biogeographical regions (Figure 3c, *t* = 24.5077, *p* < .001). Nestedness showed an opposite pattern, being significantly higher within than across biogeographical regions (Figure 3b, *t* = 15.708, *p* < .001). Similar patterns can be also observed in the pairwise comparison among biogeographical regions (Table 1). The trend of the Jaccard dissimilarity and its turnover and nestedness components showed consistent patterns. However, the *t* test did not support significant differences between the Mediterranean and Continental biogeographical regions for both turnover and nestedness components. The robustness of these results was confirmed by the analyses performed with independent subsets of sites (Appendix S1).

**Table 1:** Comparison of the total Jaccard dissimilarity (Beta Dissimilarity) and its turnover and nestedness components within and across each biogeographical region for each pair of biogeographical regions

	Mean beta within	Mean beta across	*df*	*p*‐value
ALP versus CON				
Beta dissimilarity	0.910	0.937	5,834.548	<.001
Turnover	0.855	0.904	5,851.924	<.001
Nestedness	0.055	0.033	5,801.476	<.001
ALP versus MED				
Beta dissimilarity	0.918	0.954	8,765.344	<.001
Turnover	0.873	0.929	8,852.728	<.001
Nestedness	0.045	0.025	9,036.273	<.001
CON versus MED				
Beta dissimilarity	0.912	0.907	4,325.297	<.01
Turnover	0.877	0.881	4,357.295	N.S.
Nestedness	0.035	0.036	4,198.186	N.S.

Abbreviations: ALP, Alpine region; CON, Continental region; MED, Mediterranean region.

## 4 DISCUSSION

Our results provide only partial support to the consistency of the biogeographical regionalization of Italy. While the differences in forest plant communities clearly support the distinction between the Alpine and the other two regions, differences between Continental and Mediterranean regions are not supported, despite the fact that the comparisons were performed within a comparable extent. As expected, pairwise beta‐diversity and its turnover component are higher between than within biogeographical regions, even after controlling for spatial extent (Nekola & White, 1999; Palmer, Earls, Hoagland, White, & Wohlgemuth, 2002). This indicates that different regional species pools contribute to the assembly of local communities (Carstensen et al., 2013) and that spatial distance across biogeographical regions has a stronger effect than within biogeographical regions. In general, the nestedness component plays a minor role in structuring forest plant communities, probably due to the heterogeneity of the forest types that were selected by the probabilistic sampling design that was adopted in our study. However, nestedness is slightly greater within biogeographical regions, likely indicating that local habitat conditions responsible for differences in species richness are more important than between biogeographical regions (Carstensen et al., 2013; Jiménez‐Alfaro et al., 2018). These findings support the existence of a biogeographical structure of the species pools at broader spatial scales (Cornell & Harrison, 2014; Karger et al., 2016) that is captured by the biogeographical regionalization (Jiménez‐Alfaro et al., 2018) and by the assembly of local plant communities. However, a low differentiation in turnover and nestedness, as well as in composition, between the Mediterranean and Continental biogeographical regions suggests that the biogeographical regionalization is not well supported.

Besides the role of the Alps as topographic barrier, which limits the dispersion of species, and the low connectivity between Alps and Apennines, the distinctiveness of forest plant communities in the Alpine region is certainly related to a strong latitudinal and elevation difference compared to other Italian regions where distinct climatic differences are more important. Climatic factors, related for instance to the water–energy balance (Hawkins et al., 2003; Whittaker, Nogués‐Bravo, & Araújo, 2007), are among the main drivers of biogeographical patterns (Mucina, 2019; Rueda et al., 2010; Sexton, McIntyre, Angert, & Rice, 2009), influencing fundamental biotic processes as reproduction, dispersal, and establishment. According to the climate hypothesis (Hawkins et al., 2003), these processes play a key role in controlling species distribution patterns. In this perspective, both latitude and elevation are likely to contribute to the climatic and biotic uniqueness of the Alpine region. These geographical and physical features had also influenced historical dynamics. In particular, historical differences between the Alpine and the other two regions were mainly determined by the dynamics of Quaternary glaciations. While the Alps were almost completely covered by glaciers, with only some glacial refugia in the peripheral parts of the chain (Schönswetter, Stehlik, Holderegger, & Tribsch, 2005), the rest of peninsular Italy was almost ice‐free, except for some scattered areas along the Appennines (Hughes, Gibbard, & Woodwar, 2006). This historical phase strongly influenced biogeographical and evolutionary processes, determining species distribution patterns across Italy and contributing to the distinctiveness of the Alpine biota (Schönswetter et al., 2005). These historical aspects would confirm the idea that biogeographical regions are dynamic entities over evolutionary time (e.g., Mucina, 2019).

The distinction between the Continental and Mediterranean regions is not supported by our results. The observed higher beta‐diversity within than between these regions and the nonsignificant differences in turnover and nestedness patterns, or in community composition, would suggest a prudent use of this biogeographical regionalization, at least for the Italian peninsula. This could be due to the fact that forests of these regions are mainly distributed along Apennines, a geophysical homogeneous structure that connects both northern and southern regions of the Italian peninsula, as well as the Adriatic and the Tyrrhenian sides. Our probabilistic sample of the Italian forests is consistent with a highly connected system where the virtual absence of dispersal barriers and the presence of a comparable past history (Vacchiano, Garbarino, Lingua, & Motta, 2017) determined a strong compositional overlap among the forest plant communities of these two biogeographical regions. Therefore, Apennines could represent a biogeographical transition zone (Morrone, 2018).

Our results demonstrate that an analytical evaluation of the variation in species composition among regions is a promising approach for testing the consistency of biogeographical regionalization models (Morrone, 2018). In particular, the analysis of beta‐diversity and its constitutive components (i.e., species turnover and species nestedness) proved to be an effective way to examine community patterns across and within regions. This approach could therefore provide the ecological background support to the biogeographical regionalization used in some environmental conservation polices adopted by EU, where an analytical validation is actually lacking. However, we are aware that diversity patterns may be also habitat and taxon specific. For example, forests have experienced a long history of transformation where ecological and management‐driven processes interacted with biogeographical processes in shaping community assemblages (Svenning & Skov, 2005). Other less‐disturbed biota, such as high elevation grasslands or outcrops, could instead maintain a stronger biogeographical footprint (Soininen, McDonald, & Hillebrand, 2007). The same can be stated considering the distribution patterns of less mobile organisms. In contrast, dynamics in more impacted habitats (e.g., in agroecosystems) or dynamics of mobile organisms (e.g., spore dispersing fungi) could be less sensitive to biogeographical processes being more prone to homogenization (Winter et al., 2009). This claims for further large scale, multi‐habitat, and multi‐taxon studies and for a habitat and taxon‐specific approach to conservation (Burrascano et al., 2018; Flensted et al., 2016; Nascimbene et al., 2013). According to these findings, EU conservation policies should distinguish between habitat and taxa that are manageable according to a biogeographical approach (McIntosh & Burbidge, 2014) and those that are more influenced by local processes. This would therefore require a more context‐specific approach.

## CONFLICT OF INTEREST

None declared.

## AUTHOR CONTRIBUTIONS

Alessandro Chiarucci and Juri Nascimbene are plant ecologists whose main research activity focuses on the exploration of community biogeographical patterns at multiple spatial and temporal scales and on biodiversity conservation. A.C., J.N., M.D., R.C. conceived the ideas; R.C., G.C., S.C. coordinated the data assembly; S.L., D.G., C.L. analyzed the data; J.N. led the writing. All the authors contributed to writing.

## Data Availability

Data supporting this paper are included in the ICP Forest Collaborative Database “LI‐BioDiv” (based at Program Co‐ordinating Centre in Eberswalde, Germany). Data on vascular species by forest type and biogeographical region are available from the data repository Dryad, https://doi.org/10.5061/dryad.qh78q82.
