# Supplementary material for: Exploring patterns of beta‐diversity to test the consistency of biogeographical boundaries: A case study across forest plant communities of Italy
Source: Ecol Evol. 2019 Oct 2;9(20):11716–23. doi: 10.1002/ece3.5669 (PMC6822039; doi:10.1002/ece3.5669)
Supplement: Supplementary file 1 [file ECE3-9-11716-s001.docx]

**Appendix 1**

To quantify the risk of biases due to non-independence of the values in pairwise beta, turnover and nestedness analyses, we repeated the analyses considering two independent randomly selected subsets of plot data from each of the three regions. Each subset includes 50% of the total number of sites of each region. We calculated total Jaccard dissimilarity (Beta Dissimilarity) and its turnover and nestedness components within and between regions using the two different subsets for each region (i.e., the 50% of the plots of the Mediterranean region used to calculate beta/nest/turn within the region were excluded from the analysis between regions). We, then, applied an unpaired *t*-test between the pairs calculated within and between biogeographical regions. We replicated the analyses 100 times and we obtained the percentage of significant differences. The results are shown in the table below, which corresponds to the Table 1 included in the manuscript. Even decreasing the size of the sample, results remain the same (the only minor difference is the non-significance of the total beta diversity between Mediterranean and Continental region) that are reported and discussed in the manuscript.

**Table** Comparison of the total Jaccard dissimilarity (Beta Dissimilarity) and its turnover and nestedness components within and across each biogeographical region, using two independent subsets of plot data randomly extracted from each region. ALP: Alpine region; CON: Continental region; MED: Mediterranean region. P-value<0.001 (%) indicates the percentages of significant differences on a total of 100 replicates.

|  | Mean Beta Within | Mean Beta Across | df | P-value <0.001 (%) |
| --- | --- | --- | --- | --- |
| ALP vs CON subset |  |  |  |  |
| Beta dissimilarity | 0.914 | 0.938 | 1543.652 | 99 |
| Turnover | 0.861 | 0.904 | 1540.3 | 99 |
| Nestedness | 0.053 | 0.032 | 1492.652 | 90 |
| ALP vs MED subset |  |  |  |  |
| Beta dissimilarity | 0.923 | 0.963 | 2888.67 | 100 |
| Turnover | 0.881 | 0.944 | 2863.97 | 100 |
| Nestedness | 0.042 | 0.02 | 2587.3 | 100 |
| CON vs MED subset |  |  |  |  |
| Beta dissimilarity | 0.918 | 0.916 | 1522.393 | 33 |
| Turnover | 0.886 | 0.879 | 1484.952 | 34 |
| Nestedness | 0.033 | 0.035 | 1457.255 | 32 |
